# Exploring links between resilience and the macro-level development of healthcare regulation- a Norwegian case study

**DOI:** 10.1186/s12913-020-05513-x

**Published:** 2020-08-18

**Authors:** Sina Furnes Øyri, Geir Sverre Braut, Carl Macrae, Siri Wiig

**Affiliations:** 1 Faculty of Health Sciences, SHARE - Centre for Resilience in Healthcare, University of Stavanger, Stavanger, Norway; 2 Stavanger University Hospital, Stavanger, Norway; 3 Centre for Health Innovation, Leadership and Learning, Nottingham University Business School, Stavanger, Norway

**Keywords:** Quality improvement, Patient safety, Resilient performance, Adaptive capacity, Quality improvement regulation, Performance-based regulatory regimes, Governmental bodies, Regulators, Management, Implementation, Involvement

## Abstract

**Background:**

The relationship between quality and safety regulation and resilience in healthcare has received little systematic scrutiny. Accordingly, this study examines the introduction of a new regulatory framework (the Quality Improvement Regulation) in Norway that aimed to focus on developing the capacity of hospitals to continually improve quality and safety. The overall aim of the study was to explore the governmental rationale and expectations in relation to the Quality Improvement Regulation, and how it could potentially influence the management of resilience in hospitals. The study applies resilience in healthcare and risk regulation as *theoretical perspectives.*

**Methods:**

The *design* is a single embedded case study, investigating the Norwegian regulatory healthcare regime. Data was collected by approaching three regulatory bodies through formal letters, asking them to provide internal and public documents, and by searching through open Internet-sources. Based on this, we conducted a document analysis, supplemented by interviews with seven strategic informants in the regulatory bodies.

**Results:**

The *rationale* for introducing the Quality Improvement Regulation focused on challenges associated with implementation, lack of management competencies; need to promote quality improvement as a managerial responsibility. Some informants worried that the generic regulatory design made it less helpful for managers and clinicians, others claimed a non-detailed regulation was key to make it fit all hospital-contexts. The Government expected hospital managers to obtain an overview of risks and to *adapt* risk management and quality improvement measures to their specific context and activities.

**Conclusions:**

Based on the rationale of making the Quality Improvement Regulation flexible to hospital context, encouraging the ability to anticipate local risks, along with expectations about the generic design as challenging for managers and clinicians, we found that the regulators did consider work as done as important when designing the Quality Improvement Regulation. These perspectives are in line with ideas of resilience. However, the Quality Improvement Regulation might be open for adaptation by the regulatees, but this may not necessarily mean that it promotes or encourages adaptive behavior in actual practice. Limited involvement of clinicians in the regulatory development process and a lack of reflexive spaces might hamper quality improvement efforts.

## Background

International studies show that despite significant efforts, quality and safety in healthcare remains a major challenge, and adverse events rates among hospitalized patients are still high [1, 2]. Some of the fundamental challenges in quality and safety are related to how organizations are led and managed, particularly in relation to improvement activities, with a recent progress report calling for stronger leadership commitment and acknowledgment of quality and safety as integral to the operational culture of healthcare organizations [3]. Investigations into major healthcare failures, such as the Mid Staffordshire and Morecambe Bay inquiries in the UK, found issues with poor management and organizational oversight of safety [4, 5]. One important issue is therefore how regulators should try and address issues of organizational leadership, engagement and management of patient safety [6, 7].

Previous research from the British National Health Service reveals a vast number of guidelines and standards that clinicians are expected to comply with, which can create practical challenges and difficulties in identifying the most relevant or essential rules [8]. Equally, there are concerns that the complexity and demands of external regulation might distract organizations rather than support them in efforts to improve quality and safety [7]. Therefore, it is important to explore how regulators seek to shape and co-opt organizational activities to effectively manage and improve quality and safety. The complexity and variation in healthcare means it can be challenging—and at times impossible—to provide detailed rules and regulations that adequately fit every context. Thus, regulatory approaches that support flexibility and local adaptation can be useful, if not essential [9–12]. Understanding flexibility and adaptive capacities is a central concern of the field of resilience in healthcare, where much recent work has attempted to conceptualize the adaptive processes and resilient capacities that underpin quality and safety in complex settings (see Table 1 for conceptual clarifications) [3, 21, 22].

**Table 1:** Conceptual Clarifications

**The relationship of Quality, Safety and Resilience**	
• Different paradigms exist when it comes to resilience. This paper relies on a resilience engineering tradition that has been applied in healthcare [13]. • There is not always a clear distinction between the concepts of quality and safety in healthcare. • According to the Institute of Medicine, and the Norwegian adoption of the conceptualization of quality, quality consists of six dimensions: clinical effectiveness, patient safety, patient centeredness, care coordination, efficiency, timeliness, and equity [14, 15, 16, 17]. • Some definitions view safety as an “attribute of quality”, and successful healthcare outcomes as results from quality efforts [18]. According to Sheps & Cardiff [18] this view misses that tradeoffs, complexity and variability are important elements in healthcare. • *In this paper,* we argue that there are different quality *dimensions* with safety as one dimension. Resilience is about creating and obtaining high quality services (Safety-II). We thus apply a wider definition compared to traditional literature focusing on risk and safety (Safety-I). Our perspective is in line with the ongoing Resilience in Healthcare Research Program (2018–2023) [12]. • We define resilience as “the capacity to adapt to challenges and changes at different system levels, to maintain high quality care” [12].	
**Resilient** ***Performance***	
• According to Hollnagel [19], any organization that manages to respond to, monitor, learn from and anticipate both expected and unexpected events would in a strict sense have *potential* for resilient performance. Performance, however, is complicated to study and to measure theoretically because it depends on context and local circumstances. • *In this paper,* the potential for resilient performance is explored as the potential to adapt regulatory requirements into daily work practices.	
**Regulation**	
• Legal and regulatory matters are primarily developed, applied and disputed within national borders. This makes legal terminology and regulatory activities multifaceted and not easy to interconnect on an international scale. • *This paper* defines the phenomenon of regulation generally and specifically: 1. as a general governmental mechanism/instrument (including inspection; supervision). 2. as one specific Norwegian regulatory framework; regime referred to in this paper as *the Quality Improvement Regulation* with a capital “R” in “regulation”. • *In this paper* a regulatory system of *Internal Control* is defined as *enforced self-regulation* characterized by the organization’s individual responsibility to apply systematic measures to ensure that all organizational activities are planned, organized, carried out and maintained in accordance with governmental requirements- and health legislation [20]. • We define *performance-based* regulation as a regulatory instrument that requires certain outcomes (achieved or avoided) without specifying any solutions [9].	

However, the traditional focus in research on quality and safety in healthcare has been on work done at the sharp-end, and less research effort has examined the detailed relationship between regulatory activities and quality and safety improvement [23–29]. Likewise, there has been limited macro-level research exploring how regulatory activities at a national level relate to resilience in healthcare. Studies on the mechanisms of resilience across multiple levels of the healthcare system are relatively rare [28–33]. Accordingly, this study seeks to explore the link between risk regulation and resilience. Specifically, it examines the assumptions and rationale that lie behind the development of a new regulatory regime in the Norwegian healthcare system, which seeks to encourage the organizational management and leadership of quality and safety improvement. This new regulatory regime is defined within the *Regulation of management and quality improvement in the healthcare services* [34], and in this paper referred to as “the Quality Improvement Regulation” (see Table 2).

**Table 2:** The Norwegian Regulatory Context*

**Size and Scale of the Norwegian Specialized Health System**	• 1,967,758 million people were treated in hospital units in 2018. • 114,028 thousand people employed in the specialist healthcare services in 2018. • 2667 EUR (27,100 NOK) in operating expenses per inhabitant in 2018.
**Governmental Regulatory and Policy-making Bodies**	• The *Ministry of Health and Care Services* directs health and care services through comprehensive legislation, annual budgetary allocations and by means of various governmental institutions such as the *Norwegian Board of Health Supervision* and the *Norwegian Directorate of Health.*
**Quality and Safety Challenges in the Norwegian Healthcare Services**	• Indications of an 11,9% adverse event rate in 2018, against 13,7% in 2017 in the hospital context. • Lack of adequate management responsibility and competencies. • Lack of competence and implementation of the *Internal Control Regulations in the Healthcare Services* developed to ensure sound professional practice and service quality and safety in the Norwegian healthcare system. • Areas of non-compliance with governmental requirements are believed to be related to hospital managers’ attitudes, values and the development of organizational culture with emphasize on learning.
**Governmental Regulatory Response to these Challenges**	• Regulators adjusted and replaced the former *Internal Control Regulation in the Healthcare Services* with the new *Regulation for management and quality improvement in the healthcare services* (hereafter referred to as: the Quality Improvement Regulation), effective from January 1st 2017. • This Quality Improvement Regulation embodies the overall aim of contributing to professionally sound practice, quality improvement and patient- and user safety, and compliance with other requirements. • It requires hospitals to plan and establish barriers in order to discover failure before it has consequences for the patients, and to handle, correct and evaluate adverse events and failures. • The focus on the managerial level and the role of leaders in risk management and quality improvement increased significantly with the new Quality Improvement Regulation. • The Ministry of Health and Care Services requests knowledge about how the hospitals comply with- and implement the new Quality Improvement Regulation.

* [1, 20, 34–41]

### Aim

The overall aim of this study was to explore:

1. how one particular country (Norway) developed a new Quality Improvement Regulation that aimed to co-opt organizational capacity to manage and improve quality and safety, and
2. in what ways regulators expected this new Quality Improvement Regulation to relate to the capacity for resilience in hospitals.

### Theoretical framework

This study drew on theories of risk regulation to explore the development and implementation of the new Quality Improvement Regulation, as well as theories of resilient healthcare, which emphasize adaptive capacities, to understand how regulators expect the new Quality Improvement Regulation to influence hospitals’ work on quality and safety.

#### Risk regulation

Laws and regulations are an essential part of how society manages risks [42]. The idea of a “risk regulation regime” seeks to explain and analyze the interacting ideas, rules and practices associated with the regulation of risks, such as the relationship between regulators and people at the front-line [43], and the role of different stakeholders such as policy makers, regulators and managers [44]. Different forms of regulatory activity can be conceptualized as a pyramid of regulatory strategies that are responsive to different degrees and forms of risk [45], with less coercive strategies at the bottom (such as self-regulation independent of government activity, see Table 1 for clarification) and more interventionistic strategies at the top (for instance, prosecution).

According to Vincent and Amalberti [46], different approaches to quality and safety can vary due to the need for standardization and control on the one hand *and* adaptability on the other. Because healthcare is complex with different types of activities and clinical settings it is not possible to rely on one “primary model” [46]. It is a demanding task to develop detailed rules and regulations that would fit many different organizational contexts, so regulators commonly leave details and specific decisions on how to manage safety and quality up to the regulatee [47]. Whereas the American regulatory system has a tradition of governing by prescriptive rules and regulations, the Norwegian system is performance-based with functional requirements, also referred to as a system of “Internal Control” (see Table 1 for detailed definition). A degree of trust is therefore required between the regulator and the regulatee, and risks are mainly handled based on norms and legal standards [9, 10, 47, 48]. The Quality Improvement Regulation we explored in this study represents such a system of enforced self-regulation and internal control, implying a performance-based approach to regulation that makes hospital management responsible for clinical performance and quality and safety.

#### Resilience in healthcare

Resilience in healthcare constitutes a valuable framework that helps to explain how systems are improved and can function despite disruptions and adverse events [49]. A core idea is that resilience is “the ability of the healthcare system to adjust its functioning prior to, during, or following changes and disturbances, so that it can sustain required performance under both expected and unexpected conditions” [13, 50]. Two approaches to safety have recently been delineated in the resilience literature. “Safety I” views safety as the absence of adverse events and failures and builds on linear processes and reactive measures [51]. In contrast, “Safety II” emphasizes the importance of focusing on what makes things go right, and that it can be hard to precisely predict and anticipate future events. The assumption is therefore that people must continually adjust and adapt to variability. Resilience is therefore regarded a key priority in healthcare [13, 50, 52] and capacities of *anticipation* (know what to expect; anticipate future developments), *adaptation* and *flexibility*, are key to understanding how healthcare organizations are capable of delivering services when challenges or disruptions occur [13, 22, 29, 53].

In this study, we specifically considered that adaptation to variation is a necessary component of safety, and that efforts to manage and improve quality depend on adaptation to local conditions and context. The degree and type of adaptation that may be required depends on the specific hospital setting and quality challenges that are being faced [46]. Berg and Aase [54] identify empirical studies of adaptive capacities at different levels. At the level of individual clinicians, adaptive capacities included dealing with unexpected situations, developing rules and procedures and improvising [54]. The ability to anticipate was found to be closely related to the ability to adapt. At the management level, “anticipatory regulation” was described as the ability to anticipate the need for resources, such as staffing levels, in line with patient demand [54].

Resilient Healthcare – Work as Imagined versus Work as Done.

According to Hollnagel [19] it is a crucial question to explore why people act the way they do: in the planning phase of work operations, the managing phase of actual work and in the phase of analysis after work has taken place (regardless of the outcome). The planned work phase is characterized by Work As one Imagine it do be (WAI), while the phase of actual work, is labeled Work As Done (WAD). Design of laws and regulations, management of quality and safety efforts including supervision, are all considered WAI. There is often an alignment challenge between WAI and WAD, that we need to explore and address to understand resilience in healthcare [19, 55].

### Research questions

The following research questions guided this study:

- What was the regulatory rationale for developing a management-focused regulatory framework (the Quality Improvement Regulation) for quality and safety improvement in healthcare?
- How do the regulatory bodies expect the new Quality Improvement Regulation to influence resilience in hospitals?

## Methods

A single embedded case study design was chosen to explore the phenomenon of resilience associated with regulation and supervision in its real-world context [56]. The *case* was defined as the regulatory design and implementation of the *Regulation on management and quality improvement in the healthcare services* and its impact on hospital managers quality and safety improvement, including the nurturing and/or hampering of resilience potentials in hospitals. The study examined three levels of healthcare oversight: governmental bodies of regulation (macro-level), regional supervision (County Governors), and hospital managers. In this article, we report on the analysis of macro-level governmental regulatory bodies.

### Data collection and recruitment

Methodological multiplicity is useful when researching complex phenomenon such as resilience associated with regulation and supervision. The data collection consisted of documents and semi-structured qualitative interviews, illustrated in Table 3.

**Table 3:** Empirical Foundation of the Study

**Documents identified, selected, read and analyzed:**	
2002	• Internal Control Regulations in the Healthcare Services (hereafter referred to as: Internal Control Regulation) [20] (2 pages)
2013	• Circula on management in hospitals, provided by the Ministry of Health and Care Services (the Ministry) [57] (5 pages) • Assignment letter of drafting a new Quality Improvement Regulation, sent from the Ministry to the Norwegian Directorate of Health (the Directorate) [58] (3 pages) • Project plan sent from (the Directorate to relevant stakeholders [59] (8 pages)
2014	• Invitation to give input to the Directorate’s draft of the new Quality Improvement Regulation [60] (2 pages) • Draft of the Hearing Memorandum sent to the Ministry, provided to them by the Directorate in cooperation with the Norwegian Board of Health Supervision (the Inspectorate) [61] (47 pages)
2015	• Final Hearing Memorandum submitted to relevant stakeholders, by the Ministry [37] (41 pages) • White Paper (NOU) [36] (344 pages, with exceptions)
2016	• Hearing Comments [62] (38 pages) • The Prerogative document for the Quality Improvement Regulation on management and quality improvement in the healthcare services, which stated the narrative of the facts and circumstances of its policies. Formal approval was given in Royal Assent [65] (4 pages)
2017	• Regulation on management and quality improvement in the healthcare services [34] (3 pages) • Guidelines relating to the Regulation on management and quality improvement in the healthcare services [66] (57 pages)
**Individual Interviews (in total 7):**	
2018	• 3 interviews at the Ministry of Health and Care Services • 2 interviews at the Norwegian Directorate of Health • 2 interviews at the Norwegian Board of Health Supervision

The three key national policymaking- and regulatory bodies in charge of developing and stimulating implementation of new healthcare regulation in Norway are the Ministry of Health and Care Services (hereafter referred to as: the Ministry), the Norwegian Directorate of Health (hereafter referred to as: the Directorate) and the Norwegian Board of Health Supervision (hereafter referred to as: the Inspectorate). Through formal letters, we requested these bodies to provide internal and public documents on the development- and implementation process of the new Quality Improvement Regulation. Internal documents, several of them exempted from public disclosure, and public documents were retrieved. These are considered legitimate sources of law [67]. Additionally, we accessed federal documents by search through open Internet sources. The documents formed the main data material in exploring the regulatory bodies’ rationale; motives and purposes for adjusting the Internal Control Regulation into a new management-focused regulatory framework for quality and safety improvement (the Quality Improvement Regulation).

After reviewing the documents, we conducted semi-structured interviews with seven informants positioned at the Ministry, the Directorate and the Inspectorate. These informants were chosen because they were recommended by our contacts in the organizations as key figures in the development process of the Quality Improvement Regulation (see the informants’ characteristics in Table 4). We recruited them to explore their considerations on the rationale and their expectations. Their educational backgrounds were a mix of economics, management, sociology, law, medicine and engineering. Informants were contacted by e-mail, informed about this study’s focus area of the specialist healthcare services, and proposed participation. Five of the interviews took place at the workplace of the interviewed person, whilst two were conducted by phone. Semi-structured interviews were conducted to explore informants’ experiences of their “world of the case” [56]. Thus, we developed a semi-structured interview guide based on theoretical perspectives on resilience and risk regulation regimes and based on information retrieved from the documents (see Supplementary file 1). The topics included: rationale, experiences of stakeholder involvement and information processes, expectations regarding implementation and capacity for regulatory flexibility. Moreover, the semi-structured interview guide enabled the researcher to ask additional questions based on the informant’s answers. Interview duration varied between 1 h and 1 h and 30 min. Author SFO conducted, tape-recorded and transcribed all seven interviews.

**Table 4:** Informants’ Characteristics

Informant	Background	Governmental Role
Informant 1	Economy, Quality Improvement in Healthcare	Leader
Informant 2	Health Professional, Administration in Healthcare, Quality Improvement in Healthcare	Advisor
Informant 3	Quality and Safety in Healthcare	Advisor
Informant 4	Legal Professional, Administration in Healthcare	Leader
Informant 5	Health Professional	Leader
Informant 6	Engineering, Administration in Healthcare	Leader
Informant 7	Health Professional	Leader

### Data analysis

Prior to conducting the interviews, SFO read and inductively analyzed the documents, to gain an overview of the regulatory process [68]. Due to SFO’s cross-disciplinary background (Master in Law and MSc in Risk Management and Societal Safety), documents were read in terms of both directed content analysis [69] and legal dogmatic [70]. The latter aims for identifying the legislator’s meaning through textual- and contextual interpretation. The interview data was partly analyzed inductively by identifying concepts within resilience in healthcare [71], and partly deductively by using predetermined questions explicitly exploring resilience capacities. The interview transcriptions were checked for methodological quality in accordance with the consolidated criteria for reporting qualitative research [72]. We analyzed the interview data inspired by a qualitative content analysis [73]. We identified all meaning units, condensed these, identified codes and sub-categories. Sub-categories were formed in line with the resilience capacities of anticipation, adaptation, flexibility. Finally, we sorted the sub-categories into themes, summarized to reflect the perspectives of “rationale” and “expectations” according to the research questions. Researcher SFO led the analysis process, while GSB and SW read the interviews and contributed in discussion about the results, developing and refining the categories.

## Results

The results from documents and interviews were analyzed separately, but are presented together*,* and described theme-wise. This structure was chosen because most of the informants played some part in the regulatory development process by either contributing in writing the Quality Improvement Regulation or issuing the Hearing Memorandum (containing suggestions and draft of the Quality Improvement Regulation), the Prerogative document approving the Quality Improvement Regulation [74], Hearing Comments or Guidelines. In Table 5 we summarize the themes, subcategories and main findings.

**Table 5:** Themes, Sub-Categories and Key “take home” Points

**THEME-I RATIONALE**	
**Sub-Category**	**Key Points**
Adaptation & Flexibility	The new Quality Improvement Regulation was elaborated and adapted to meet the needs from the services: • Modernized by adding management and quality improvement • Designed around a PDSA structure • The obligation to delegate tasks in daily work was specified • One new substantial provision was added: The obligation to systematically evaluate risk management and quality improvement measures (yearly) The Quality Improvement Regulation per se is flexible in its non-detailed, regulatory design, because: • The rules can be adapted to any hospital organization
**THEME-II EXPECTATIONS**	
**Sub-Category**	**Key Points**
Adaptation & Flexibility	The Government expected hospital managers to: • implement risk reducing- and quality improvement measures based on specific context, size, activities and risk picture Design-wise, the Quality Improvement Regulation may be flexible as it leaves the regulatees to decide on details for implementation, but: • this does not necessarily mean that it encourages adaptive behavior in actual hospital work practices • it is challenging to make the Quality Improvement Regulation relevant for the right clinical level The Government did: • not have a clear vision of how hospital managers would adapt it to their practical work • suspect a disconnection between what the top-level managers prioritize and what is done at the level where clinical work unfolds
Anticipation	The Government expected hospital managers to: • obtain an overview of- and reveal risk factors prior to failure

### Theme I - governmental rationale for revising the quality improvement regulation

#### Modernization - language and appeal

The documents by the Ministry [37, 38] described how a regulatory revision was important due to lack of compliance and a need to unite the previous internal control regime with systematic quality and safety improvement. The interviewed informants highlighted discontent with the former Internal Control Regulation. Overall, they perceived the control component to not sit well within the field of clinical practice. They claimed the term “internal control” was an alienating term that was too technical. Several institutions in the hearing process therefore agreed to exclude the term “internal control” from the new Quality Improvement Regulation. Some informants claimed that the former Internal Control Regulation was bureaucratic, blaming its non-pedagogical design and inaccessible language. All informants pointed to a need for modernization, adaptation, simplification and better explanation in the Quality Improvement Regulation. Informants believed there had been limited success in making the former Internal Control Regulation a “living document”. Governmental documents listed risk management and leadership requirements, coordination of services and causal analysis of adverse events as important areas to clarify in a new Quality Improvement Regulation. Elements of management responsibility, co-worker involvement and quality improvement were specified and promoted in the new Quality Improvement Regulation. Some informants described problems of under-communication to hospital leaders, making the former Internal Control Regulation less known than it could have been.

“They did this in an overly bureaucratic and wrong way with a lot of emphasis on written procedures and things like that (…) it seemed very alienating, so you couldn’t get the rationale [of the former Internal Control Regulation] (…) and selling the idea was very difficult, many who simply did not understand it” –Informant 5

#### A shift in focus: leaders’ responsibility for quality improvement and co-worker involvement

The findings indicated that including “management” in the title of the Quality Improvement Regulation would emphasize the importance of hospital managers in the continuous improvement of quality and patient safety. Some informants noted that management challenges existed in general in the healthcare services, and all highlighted the lack of focus on leadership and management elements in the former Internal Control Regulation. Both the documentation produced by the Inspectorate, and informants referred to their own experiences retrieved from supervision when they argued for a stronger and specified management-focus. The Quality Improvement Regulation’s title words “management” and “quality improvement” appealed to people in the healthcare services, our informants argued.

Several informants described the Quality Improvement Regulation’s *mandate* to hospital leaders as an advantage. According to The Prerogative document approving the Quality Improvement Regulation [65], the judicial accountability for implementing and governing a management system for quality and safety lies with the hospital’s CEO. The Directorate [64] however, argued against a provision that solely focused on the overall responsible leader and urged the Ministry to consider including a provision that stressed that leaders at every level of healthcare organizations have responsibility to assure compliance and be responsible for the requirements in the Quality Improvement Regulation. However, to avoid uncertainty over the formal, top-level management responsibility, such a provision was not included. Nonetheless, the responsibility to implement certain tasks and make these operational at department- or unit level, may be delegated by the hospital leader, meaning that employees in the entire organization are expected to be involved in the quality improvement process, ref. § 3 in the Quality Improvement Regulation,

“Anyone who has overall responsibility for the organization shall ensure that systematic management of the organization's activities is established and implemented in accordance with these regulations and that the employees of the organization contribute to this”.

Informants considered the term “contribute to” important, because co-workers are familiar with the daily challenges and are often best placed to improve the quality of clinical systems. Indeed, the Guidelines developed for the Quality Improvement Regulation acknowledge that leaders close to clinical work are often the ones who practically implement quality improvement measures in large organizations [66].

#### Quality improvement in accordance with the systematic PDSA approach

The Quality Improvement Regulation categorizes and explicate the duties of planning, implementation, evaluation and correction in line with Deming’s [75] “Plan, Do, Study, Act”- cycle (PDSA). These obligations are extensive, and according to provision §5 all four categories of duties presuppose an overview of the organization’s activities, structures, competences and risks, including how to develop and improve routines and tasks. The Directorate’s documentation [64] described how they expect that a PDSA structure would give the Quality Improvement Regulation a more educational approach, stressing how the link between the different provisions then would appear clearer (provide more information to support and educate people). Based on positive feedback relating to the inclusion of quality improvement principles drawn from Deming’s [75] work that was included in the former Internal Control Regulation, there was agreement on retaining that logic in the new Quality Improvement Regulation, as long as the systematic quality improvement approach was elaborated in more detail. Although the Inspectorate [63] described the PDSA approach as “an exciting idea”, they argued that the model would lead to several disadvantages if not every four PDSA steps were distinguished into equivalent four separate provisions in the Quality Improvement Regulation. Informants noted that quality improvement - and patient safety work tend to be for enthusiasts only and supported using Deming’s four phases. It would make it easier to remember and relate to, especially for people with no legal background, they argued.

“… people have started to get used to that way of thinking [PDSA]. So, we thought they might recognize themselves if having these four elements [in the Quality Improvement Regulation]. We tried to write it in a comprehensive way with management focus and enhance or clarify that”. ¨

–Informant 4

#### Regulatory design adjustments

Overall, the informants expressed the need for new legal adjustments aiming at quality improvement and patient safety measures. Our documents indicated that from a strict legal point of view, the former Internal Control Regulation and the new Quality Improvement Regulation more or less overlap, but one new substantial provision was included: the obligation to evaluate risk management and quality improvement measures systematically once a year.

Regardless of similar legal aims, informants described the new Quality Improvement Regulation as moving away from an Internal Control Regulation they referred to as having a “static” design, towards a more “dynamic” approach. They believed the Quality Improvement Regulation had a better balance between reactive and proactive approaches.

“The former Internal Control Regulation seemed a little static, seemed a bit like the intention of making a book, “job done”, while this (new) one is probably more dynamic, I think that is the idea”.

- Informant 7

Some of the informants described how the Ministry strived in their initial effort to make an integrative regulation, applicable for both specialist healthcare services (responsible for the hospital sector), and the municipalities (responsible for primary care services: general practitioners, nursing homes, home care, ambulatory care) in Norway. The main challenge was related to making sure that the Quality Improvement Regulation was applicable at all system-levels. Some informants described an internal battle within the government institutions, led by The Ministry of Local Government and Modernization, which did not wish to regulate in detail, but instead wished to have a regulation with the capacity of being adaptable to the size of the individual entity. One informant wished the Quality Improvement Regulation covered even more than it ended up doing, arguing that the lawyers were not willing to accommodate this. According to the documentary evidence, the Quality Improvement Regulation ended up having a relatively broad overall design, to allow it to fit all sorts of healthcare organizations. Several of the informants pointed towards the advantage of underspecifying the Quality Improvement Regulation, because it forces the managers to think about what is applicable and required in their context, adapting the rules to the organization and their entity. One informant argued that while the Quality Improvement Regulation is more detailed than other regulations, it is however “deadly to become too detailed”.

“It is a system for robustness (…) without forcing it on people”.

- Informant 1

On the other hand, informants' supervisory experiences showed that the overall design might lead the organizations to think the Quality Improvement Regulation is too generic: implying that the hospitals wanted the inspectors to tell them how to apply it to their context. Our findings indicated that the government therefore saw the need to provide examples in the Guidelines, to make it comprehensible for people “who do not love regulations”.

### Theme II - expectations of resilient capacities

#### Anticipation - risk as a fundamental principle

The Memorandum (containing suggestions and draft of the Quality Improvement Regulation) [37] outlined the obligation for managers: to gain an overview of risk areas, to plan prevention of risks, to reveal risk factors, with an expectation of systematic implementation of improvement measures. Managers should pay special attention to activities or processes in areas where failure or breach may occur more frequently than accepted, and in areas with potentially severe or unfortunate consequences for patients and users [66]. And it was considered important for managers to identify risk in connection with: planned changes; repeated observations of risks in relation to a specific activity; and adverse event in one part of the healthcare service which may be related to other departments or units [66].

Informants described that the new PDSA structure of the Quality Improvement Regulation allowed it to relate to everyday hospital practice, because each step of the PDSA was elaborated in the new regulatory framework. Moreover, the revised language made it much more meaningful compared to the former Internal Control Regulation. This would in turn enable the organizations to uncover real risks, they argued. Informants expected the Quality Improvement Regulation to be a potential catalyst for the hospital managers to gain a bird’s eye perspective on the risks, promoting the ability to address local risks. Some pointed out the need for change in how people within the healthcare system perceive quality improvement and for greater congruence between management and healthcare practitioners’ perspectives. They worried that the regulatory- and safety management system can become so complicated and complex that it is counterproductive to effective risk management. Some expected the Quality Improvement Regulation to be worthless if not incorporated into- and helped shape the organizational culture and management. In order to create anticipatory systems, time and resources are key, informants argued.

#### Adaptation to context

In the documentary evidence, the Ministry [37] explained that all four PDSA-steps were supposed to be connected to one another, by incorporating continuous evaluation along with relevant corrections. Both the Hearing Memorandum [37] and the Quality Improvement Regulation (§5) describe that organizations are expected to base their risk management and quality improvement measures on proportionality; that is, their own specific context, size, activities and risk picture. Similar factors should guide how the organizations choose to document and implement their measures. According to the Quality Improvement Regulation, management systems are expected to encompass deviations and adverse events. However, the documents highlighted that the type of follow up should vary according to the type of the deviation or adverse event. The Inspectorate suggested that the Quality Improvement Regulation needed a more thorough specification of how organizations should be obliged to follow-up severe adverse events, while the Ministry argued that increasing the level of detail could actually narrow the scope and applicability of the Quality Improvement Regulation. Ultimately, the final Quality Improvement Regulation did not specify *how* the organizations should comply.

In line with the Memorandum [37], many informants stressed the importance for the rules to be adapted to the hospital’s size, complexity and risks. The informants did see more advantages than disadvantages with the Quality Improvement Regulation encouraging these adaptive capacities. The Quality Improvement Regulation provides room for flexibility, depending on how it is adapted to a specific organization. However, there was some concern that top-level hospital managers would solely focus on claims; regulatory details provided by the Ministry which might lead to a lack of local adaptive capacity.

“whether you have a small organization with few employees or a large hospital with many employees, will make a difference to what risk reducing measures you implement. I do think it is an advantage that the Quality Improvement Regulation is non-detailed because it forces the managers to think about what fits their organization”

- Informant 6

#### The practical relevance for healthcare professionals

The Directorate [64] stressed its support for a more pedagogically approach in the Quality Improvement Regulation and suggested an elaboration on the four quality improvement steps (PDSA), as this could work as a checklist for managers. This would make the Quality Improvement Regulation more practically applicable especially when coupled with a set of well-prepared guidelines accompanying the Quality Improvement Regulation.

In its Hearing Comment, the Inspectorate [63] referred to the concept of resilience and research on resilience engineering, emphasizing the lack of focus on positive outcome and well-functioning processes. Thus, the Inspectorate stressed that it would be important and relevant in both the Quality Improvement Regulation and associated Guidelines, to mention the two sets of paradigms of Safety-I and Safety-II. The reasoning was this could encourage the hospitals to refer to successful experiences and activities upon which to develop relevant quality improvement measures. The Ministry [37] highlighted the importance of creating an organizational culture where results and experiences should be shared both within and cross-sector. This suggestion of tying resilience in healthcare to practical learning was not present anywhere else in the governmental documents and informants only tangentially considered this link:

“I do not remember that the concept of resilience was discussed when we planned the Quality Improvement Regulation. We had heard of resilience in other arenas, but I did not link the two processes together”.

- Informant 7

“The latest fashion to learn from successful stories, like the concept of resilience: to strengthen what is good. It makes it more proactive than to just repair. I have not thought about until now, but I think there is an improvement potential”.

– Informant 1

All the informants highlighted how demanding it was to relate the former Internal Control Regulations to practice because people across the healthcare system understood and interpreted it in very different ways. There were conflicting expectations regarding the new Quality Improvement Regulation’s applicability in practice. Some argued that it should not be difficult to move from the written text, to recognize and apply it in a specific hospital context, without interpretation. Others expected it to be difficult, wondering to what extent the Quality Improvement Regulation would be helpful. They stressed how it is not a given that all managers recognize their own role and obligations in the Quality Improvement Regulation and realize what efforts to put into practice. Interview findings suggested that it could be hard for healthcare professionals to comprehend what an underspecified regulatory design really encompasses, since no one knows the exact level of effort and measures expected. A minimum level of regulatory detail could therefore guide the healthcare service to find its own weaknesses. Some informants were quite negative towards the practical implications. They described that many people were still not fully aware of the new Quality Improvement Regulation and its content, even though governmental expectations were more explicitly expressed now compared to in the former Internal Control Regulation. The Inspectorate informants described experiences from supervision, claiming that the Quality Improvement Regulation aims to become a management tool, but it fails in becoming a *real, practical* tool:

“I think that from the authorities’ point of view, the Quality Improvement Regulation was thought to represent a “living” tool, closer to the clinical environment. I believe that it has not succeeded with that”.

- Informant 7

“It does seem a little clearer, more visible that the Quality Improvement Regulation in a sense is a management tool, but that does not imply that it is easy to get it into the practical field”.

- Informant 3

Informants confirmed that the Guidelines became a large, comprehensive document, reducing its utility and practicality.

“What I am a little worried about is that you come up with a new Quality Improvement Regulation, and then there is excitement, this is useful and so on, and then you may not have the good tools to put this into practice. Maybe five years will pass, and the national audit shows that it is not implemented (…). And, then the enthusiasm falls. So, we must work a lot more with enabling managers to meet the requirements”.

- Informant 3

Interviews indicated that high ranked policy makers did get a lot of positive feedback, both in informal and formal settings, indicating that the Quality Improvement Regulation was welcomed and perceived as useful. One informant referred to different lawyers who recounted how well received the Quality Improvement Regulation was, but noted ironically, “I would wait to pop the champagne until I see the effect in real life”. Some informants described the challenge of making the Quality Improvement Regulation relevant for the right stakeholder level. They anticipated depressing response from hospital departments if they had asked about the practical effect of the regulatory adjustments. They were curious to see the response and how “alive” the Quality Improvement Regulation was, questioning if the Ministry was too far away from the service to receive any negative response. Some described that there was no need for more rules and regulations.

“Authorities might become a little too theoretical in relation to those who work in practice and are in the middle of the patient flow and only do their job as best they can, without necessarily thinking that ‘yes, this fits section six of that regulation’”. – Informant 6

The expectations about the implementation of the Quality Improvement Regulation was the most debated and complicated aspect of the development process. There was agreement in the Prerogative document approving the Quality Improvement Regulation [65] and among informants, that the new Quality Improvement Regulation had greater appeal the way it was designed. However, most informants did not have a clear vision of how people would implement it in their practical work. In line with this, some described how there often is a disconnection between what the top-level managers prioritize and what is actually done at the level where practical work unfolds, and that practice does not change solely due to government information. Based on prior experiences where the complexity of implementation had been underestimated, the Directorate [66] laid out their expectations for the top leader and leaders at every level, to pay special attention to practical implementation efforts.

“Clinicians never disagree on the measures; it is the implementation that creates discussion and frustration because that is the hard part. (…) There is a major gap between what we do know and what we do. (…) To change practice, that is the difficult bit. (…) Dissemination of knowledge is hard work. Sometimes it happens pro forma, “we have done it”, and we can see that they have not done anything at all”.

– Informant 1

#### Support for implementation

In accordance with governmental task delegation, the Directorate is responsible for administration, information and interpretation of regulations, including the new Quality Improvement Regulation [76]. Our findings however, showed that an implementation plan for the Quality Improvement Regulation, did not exist. Although the Ministry is not set up to develop implementation programs, one informant said that they perhaps paid too little attention. The Quality Improvement Regulation was announced solely to the top management level in hospitals and to the hospital trusts. Findings highlighted that it would be time consuming to announce and voice the regulatory expectations throughout the system and the hospitals, partly because, “it takes a long time to get people to realize what they really should be doing”. Although the Quality Improvement Regulation formally was distributed online through the Ministry and the Directorate’s websites, there was a lack of further diffusion and practical implementation. We found that the regulators stressed their expectation towards the hospital’s *actual, practical* implementation*,* by specifying that there is an obligation to *accomplish* plans in the Quality Improvement Regulation (§7a), not just state the obligation to plan per se [65, 66].

We found limited involvement of clinicians in the development process and a lack of involving physicians in projects for training prior to the Quality Improvement Regulation implementation. No training was aimed at leaders. Some informants, however, stressed that courses on improvement methodology were *offered* by the Directorate and that leaders were *provided with* a framework and support. The governmental expectation towards the hospitals’ ability to be in control of their activities, was described as systematic quality improvement work as part of a long-term implementation process.

## Discussions

In this article, we have explored the governmental rationale behind developing the *Regulation on management and quality improvement in the healthcare services* and expectations to how it relates to resilience. Our findings indicated that it was developed as a response to a perceived lack of adequate management competence and responsibility in the Norwegian healthcare services. One important finding regarding the governmental expectations was that our informants were not sure if there was a *specific, practical effect* from the new Quality Improvement Regulation. This illustrates the challenge in designing regulations that accommodate the gap between work as imagined and work as done. The discussion follows in line with our research questions.

### The development and implementation process

#### WAI/WAD in the regulatory development

Earlier research emphasizes the challenge of having an imbalance between regulation and practical expertise [77]. It is important to engage those working inside complex systems, experienced in recognizing risks [77]. We found little involvement of the hospitals including clinicians in the regulatory development process. There is little evidence that the government engaged with clinicians who disagreed on the suggested quality improvement measures, but who then subsequently experienced implementation challenges. This points to the need for “reflexive spaces” to discuss and align the perspectives of policy makers, regulators, managers, and clinicians [78]. Reflexive spaces provide arenas for the involvement of “clinical, sharp end” healthcare professionals, quality advisors and hospital managers in dialogue and productive conversations about practical experience, processes and activities. In turn, policy makers and regulators are able to learn about the practical challenges of quality improvement. Moreover, by facilitating “communities of practice and storytelling” these arenas can reveal the adaptive capacity that often is present implicitly in daily works practices [79]. Lack of stakeholder involvement could contribute to regulations becoming less useful and applicable in practice, increasing the dissonance between WAI and WAD [13, 19, 80]. Our findings indicated that the government expressed awareness of this gap, which highlights an under-explored potential in designing regulations for complex healthcare environments: to deeply involve clinical managers in the design of regulatory regimes. Such co-regulatory models will provide stakeholders with a greater opportunity to make their voices heard. More broadly, it is important to create spaces for collaboration to improve quality and safety in the healthcare system [12, 78]. It may therefore be beneficial for regulators to invite stakeholders into the development and evaluation process, in both formal and informal settings, enabling knowledge exchange and enhancement between actors at the macro- and micro levels.

#### The potential for flexibility in the development of a performance-based regulatory regime

As long as the regulatory regime has capacities to adapt to different situations, anticipation is safeguarded [81]. Our analysis indicated that performance based regulatory systems inherently aim to engage with and co-opt the practical expertise of managers (managers determine or delegate specific risk- and quality improvement measures), and by supporting local flexibility and adaptation this creates a space for resilient performance.

Many studies view the gaps between WAI and WAD as a safety-issue, which therefore needs to be tightened or closed [80]. However, the perspective of Safety-II researchers is that adaptation and adjustments to local context is inevitable in healthcare [55]. Our study found that the governmental rationale for a performance-based regulatory approach was based on the assumption that adjustment and flexibility are inevitable elements of managerial and clinical work in hospital settings. This echoes a Safety-II perspective, implying that rules and regulations cannot be fully mapped and specified in advance [55]. According to Carthey et al. [8], the more that rules have a prescriptive design, the less likely workers are to comply. And, since adaptability is considered a natural human factor, full compliance is not realistic [6, 8]. This encourages room for slack and flexibility in the regulatory design. Based on our findings, we argue that the Quality Improvement Regulation supports risk-adaptive capacities by valuing context, which is a key for promoting resilience in healthcare.

One of the objectives with the proportionality principle in the Quality Improvement Regulation’s obligation to monitor performance and have oversight over risk areas, is to anticipate needs; risks and thereby adapt and adjust accordingly. Woods [82, 83] points out that data from observations and analysis of how a system adapts to *former* disruptions and adverse events, can be relevant in the assessment of the system’s potential for *future* adaptive actions. We further argue that risk analysis is a measure of which anticipation is embedded, because the rationale for analysis is to map potential risks *prior* to adverse events, in line with resilience thinking.

### Regulatory expectations

#### Adaptation

According to previous resilience research, an increased interconnected system of rules in healthcare can lead to less space for local adjustments [84]. Standards and requirements designed with little concern for sharp-end practicality can reduce the capacities of mindful local adaptation to unexpected events [30].

Vincent and Amalberti [85] describe safety as a moving target, constantly shifting with progress in innovation and prevention. Although workarounds are often developed in relation to problems in hospital environments, there is a need to develop these strategies of adaptation from local and informal improvisation into broader system-wide capacities [46]. We found an expectation of that the Quality Improvement Regulation would contribute to building adaptive capacity into the system it regulates, both prior to and when challenges and changes arise. However, if rules look good on paper, but are impossible or very difficult to translate into practice, the idea of adaptation is compromised [84]. Some informants worried that a too generic regulatory design made it less helpful for the clinicians, yet others claimed it was key to have a non-detailed regulation so it could fit all contexts. As suggested by Leistikow & Bal [11], the core regulatory challenge is thus to provide healthcare professionals with the appropriate “level of freedom to tailor quality management to their local conditions”. Further research is needed to better understand the role of hospital size and context and how different organizations use the flexibility in practice. This is an underlying principle in both the design of performance-based regulatory regimes and is core to the resilience perspective.

According to the Quality Improvement Regulation, the hospital’s CEO is expected to have formal accountability. Described as central oversight (Safety-I), this is sometimes considered to conflict with the resilient ideal of local adaptation and decentralization [86]. However, there is an option of delegation in the Quality Improvement Regulation, which was related to the capacity to adapt to varying circumstances. More specifically, we argue that the government acknowledged and expected that WAI sometimes need to be adapted to be more in line with WAD. Managers are encouraged by the Quality Improvement Regulation to adapt decisions to context, in order to meet practical circumstances such as adverse events and staffing-issues. Our findings therefore indicated that the Quality Improvement Regulation aims to facilitate a balance between the two ideals of central oversight and local adaptation. Thus, we believe this indicates that Safety-II thinking is introduced into governmental practice. This perspective is not a commonly explored assumption in the resilience- and patient safety literature, as the macro-level usually is assumed to not consider WAD and the field of practice when drafting new regulations [30, 44, 87]. Practice might be much more nuanced, and further research should focus on how other countries and regulatory systems emphasize the role of context and adaptation at the macro level.

#### Anticipation

The capacity to anticipate is characterized by foresight; to pay attention to what has not happened yet, but potentially will [19, 88]. Woods [82] describes it as looking beyond behavior in compliance with standards and norms: anticipatory aspects of resilient performance involve how people anticipate risks and bottlenecks. Organizations which emphasize proactive measures, such as monitoring, will most likely have a better potential to discover and anticipate weak signals compared to those with a less proactive approach [89]. Previous research from the Dutch healthcare system revealed that despite a complicated relationship between management and regulation of healthcare, hospitals built systems that enabled a more proactive approach to quality and safety work with the potential of facilitating innovative solutions [90]. According to our results, the hospitals have an obligation to identify- and work to uncover risks. The aspiration and intent of the regulators is that this approach will encourage anticipation. In addition, the embedded flexibility in the system could facilitate a proactive approach allowing for improvised solutions, which enables the stakeholders to anticipate these events. The Quality Improvement Regulation might foster practices that support these anticipatory capacities in hospitals and encourages awareness of for instance: risks connected to coordination of tasks and personnel, areas with a high degree of risk for failure and awareness about complaints and statistics retrieved from similar areas of activity. However, further studies are needed to evaluate the long-term effect of the implementation.

In line with Wiig and Lindøe [91], our findings revealed that the regulator has an untapped potential to engage with and obtain information from a broad range of *clinical* stakeholders. Our findings indicated that congruence between management and healthcare practitioners’ perspectives is important, which resonates with the concept of “recoupling” between different layers in the organization and the healthcare system [92, 93]. Lack of stakeholder engagement might indicate that the capacity to anticipate on a system level, is hampered. A recent study of the inspectors’ perspective on next-of-kin involvement in regulatory investigations, found that the involvement positively informed the investigations [94]. Due to the Quality Improvement Regulation, the organizations are expected to retrieve experiences and complaints from users, patients and next of kin, which can contribute in providing a learning platform for building systems with improved capacity to anticipate risks.

Previous research indicates that in systems where the regulator and the regulatee have very different roles and tasks, prescriptive regulation is even more challenging because it demands a common understanding of “adequate behavior” [6]. As discussed in this article, it seems to be a reasonable assumption that the regulatory intent is to ease that burden because the organizations are thought to possess the best knowledge on how they can improve their performance. Furthermore, it implies that what is adequate behavior in one hospital department is adequate behavior regardless of the regulator’s understanding of it. This type of a risk regulation regime emphasizes a bottom-up perspective rather than the prescriptive regime’s top-down perspective. However, we believe that it is unrealistic to expect hospitals to understand and implement legislation without pre-knowledge or assistance either from internal resources or from governmental bodies [89, 95]. If regulations are perceived as obstacles, rather than guidance, due to time- and resource consuming regulatory compliance and implementation work, regulation can compromise the ability to be flexible [89]. To promote resilience, Grote [6] suggests designing non-rigid rules specifying the underlying goals, priorities or preferred processes. Others have suggested that regulators ask organizations to demonstrate their safety management system, instead of just inspecting processes and standards [96]. By not regulating in detail, and having functional requirements, the Quality Improvement Regulation is designed to fit any healthcare organizational context. This adaptive capacity supports quality improvement measures to be more sufficiently implemented and accepted by the regulatee. Our study therefore indicates that from a resilience perspective, a performance-based healthcare system will probably be better off compared to a prescriptive one. Further exploration of these links is needed due to the limited knowledge about how interaction between system levels in healthcare influences adaptations and improvement efforts. Outputs from this study might influence how governmental bodies design and inspect rules and regulations. And interaction could fuel the goal to unite work as imagined with work as done, which favors and contributes to improve resilience capacities in the healthcare system.

### Trustworthiness and limitations

The key strength in this study is the mixed approach of traditional empirical material and legal source material, and document analysis in merge with qualitative interviews. Data was triangulated to enhance trustworthiness. Conducting the document analysis prior to the interviews helped in generating new interview questions and supplying with interviews helped avoiding “over-reliance” on documents as the sole data source [97].

This study has some limitations. The first is linguistic. **1.** “Resilience” is not in the Norwegian vocabular, neither exists a relevant translation of the term. It is fair to think that the informants used “robustness” as a way of describing anticipatory capacity, in lack of familiarity with “resilience”. However, we chose to keep the word “robustness” in our quotes from the informants. **2.** The sample size of seven interviews is a limitation but held sufficient information power due to our strategic informants who had in depth knowledge of the Quality Improvement Regulation development process and the expectations from the regulatory bodies in this area [98]. Moreover, documents were the main source of data, acting as the foundation of the Quality Improvement Regulation per se. **3.** Data retrieved from the interviews had to be taken at face value; and so was potentially exposed to the bias of informant-selective memory [99]. **4**. To ensure trustworthiness in the data analysis, three out of four researchers were involved in the analytical process of the interview material and discussed codes and sub-categories. We believe this, along with two of the co-authors’ substantial professional governance experience from the Norwegian Board of Health Supervision (author GSB) and the Petroleum Safety Authority Norway (author SW), contributed to increase the validity of our findings [56]. **5.** This paper did not aim for a complete analysis of Hollnagel’s four potentials [13, 19]. The idea of a performance-based regulatory system is to embed flexibility and as regulatory design is essential in this paper, we looked for and discussed if the regulatory design is flexible enough to facilitate adaptation- and anticipate relevant quality and safety improvement measures based on hospital context. These resilience aspects were also the focus of our predetermined interview questions. We are aware of recent critique about over-reliance on theory-founding authors [100] and partially agree. However, we believe that all Hollnagel-potentials are equally important and monitoring and learning are thus framed and discussed in a later paper (currently in review).

## Conclusions

This study’s overall aim was to explore the governmental rationale and expectations of the Quality Improvement Regulation, and how it could potentially influence the management of resilience in hospitals. Previous research identified a gap in the literature on the relationship between regulation and resilience, and to our knowledge this study will be the first to operationalize elements of resilience capacities within a regulation for management and quality improvement in healthcare. We identified a double take on adaptation: **1.** The Quality Improvement Regulation itself was adapted because the services asked for revision of the former Internal Control Regulation. This implies adaptive capacity at the macro level. **2.** Our study identified a dynamic, non-detailed regulatory framework that is expected to provide hospital managers with the potential to have risk-oversight and to adapt quality improvement measures to their organizations. Based on the Quality Improvement Regulation’s support for risk-anticipation and local adaptation, it accommodates variation in daily clinical work. The governmental rationale of making the Quality Improvement Regulation flexible to hospital context, along with regulators expectations about the overall design as challenging for healthcare practitioners, we found that the regulators did consider work as done as important when designing the Quality Improvement Regulation. These perspectives are in line with ideas of resilience. However, the Quality Improvement Regulation might be open for adaptation by the regulatees, but as our informants pointed out; this may not necessarily mean that it promotes or encourages adaptive behavior in actual practice.

There was no grand implementation plan and limited involvement of clinicians in the regulatory development process. Quality improvement efforts could benefit from inviting clinical stakeholders into the regulatory development process. Thus, we recommend the governmental bodies to co-create a plan for involvement. Moreover, with large-scale and ambitious regulatory reform such as this, it is important that the government develop an evaluation of the Quality Improvement Regulation, to map implementation efforts and activities, to explore how these have influenced quality improvement in the hospitals and to gain knowledge from how managers and clinicians experienced these activities.

## Supplementary information

**Additional file 1.** “Interview guide”. A semi-structured interview guide based on theoretical perspectives on resilience and risk regulation regimes and based on information retrieved from the documents. The topics included: rationale, experiences of stakeholder involvement and information processes, expectations regarding implementation and capacity for regulatory flexibility.

## Data Availability

Documents retrieved from online sources are publicly available. Documents exempted from public disclosure are not available. Data retrieved from the interviews is available from the corresponding author upon reasonable request and with permission from the informant(s).

## Abbreviations

The Ministry: The Ministry of Health and Care Services
The Inspectorate: The Norwegian Board of Health Supervision
The Directorate: The Norwegian Directorate of Health
The Quality Improvement Regulation: Regulation on management and quality improvement in the healthcare services
Internal Control Regulation: Internal Control Regulations in the Healthcare Services
WAI: Work as Imagined
WAD: Work as Done

## References

[1] Norwegian Directorate of Health (2019). Norwegian: Rapport. Pasientskader i Norge 2018. English: Report. Patient Injuries in Norway 2018.

[2] WHO. Patient Safety Fact File. 2019. https://www.who.int/features/factfiles/patient_safety/patient-safety-fact-file.pdf?ua=1. Accessed 8 July 2020.

[3] Gandhi TK, Kaplan GS, Leape L (2018). Transforming concepts in patient safety: a progress report. BMJ Qual Saf.

[4] Francis R (2013). Report of the mid Staffordshire NHS Foundation trust public inquiry: executive summary: stationery office.

[5] Kirkup B. The report of the Morecambe Bay investigation. 2015. https://assets.publishing.service.gov.uk/government/uploads/system/uploads/attachment_data/file/408480/47487_MBI_Accessible_v0.1.pdf. Accessed 8 July 2020.

[6] Grote, G. Leadership in resilient organizations. In: Wiig, S. & Falbruch, B., editors. Exploring Resilience. A scientific Journey from Practice to Theory. Springer Open; 2019.

[7] Oikonomou E, Carthey J, Macrae C (2019). Patient safety regulation in the NHS: mapping the regulatory landscape of healthcare. BMJ Open.

[8] Carthey J, Walker S, Deelchand V, Griffiths WH (2011). Breaking the rules: understanding non-compliance with policies and guidelines. BMJ.

[9] Coglianese C, Lazer D. Management-Based Regulation: Prescribing Private Management to Achieve Public Goals. Law & Society Review, Vol. 37, No. 4 (Dec., 2003), pp. 691–730 Published by: Blackwell Publishing on behalf of the Law and Society Association Stable URL: http://www.jstor.org/stable/1555150. Accessed 8 July 2020.

[10] Gilad S (2011). Institutionalizing fairness in financial markets: Mission impossible?. Regulation & Governance.

[11] Leistikow I, Bal RA. Resilience and regulation, an odd couple? Consequences of Safety-II on governmental regulation of healthcare quality. BMJ Qual Saf. 2020. doi: 10.1136/bmjqs-2019-010610. [Epub ahead of print]. 10.1136/bmjqs-2019-010610 PMC7553011 32229626

[12] Wiig S, Aase K, Billett S. et al. Defining the boundaries and operational concepts of resilience in the resilience in healthcare research program. BMC Health Serv Res. 2020;20:330. 10.1186/s12913-020-05224-3. 10.1186/s12913-020-05224-3 PMC7168985 32306981

[13] Hollnagel, E., Braithwaite, J. & Wears, R. L. Resilient Health Care. Ashgate Publishing Limited; 2013:xxv.

[14] Institute of Medicine. To Err is human: building a safer health system. Edited by Kohn L, Corrigan J, Donaldson M. Washington, DC: Institute of Medicine; 2000. 25077248

[15] Darzi L, Johnson A (2008). High quality care for all: NHS next stage review final report.

[16] Doyle C, Lennox L, Bell D. A systematic review of evidence on the links between patient experience and clinical safety and effectiveness. BMJ Open. 2013;3(1). 10.1136/bmjopen-2012-001570 PMC3549241 23293244

[17] Aase K (2018). Patient safety: theory and practice [in Norwegian].

[18] Doyle C, Lennox L, Bell D. A systematic review of evidence on the links between patient experience and clinical safety and effectiveness. BMJ Open. 2013;3:e001570. 10.1136/bmjopen-2012-001570. 10.1136/bmjopen-2012-001570 PMC3549241 23293244

[19] Hollnagel E (2018). Safety-II in practice.

[20] Internal Control Regulation in the Healthcare Services. In Norwegian: Forskrift om interkontroll i sosial- og helsetjenesten. FOR-2002-12-20-1731. Oslo: Ministry of Health Services; 2002.

[21] Hollnagel, E. Proactive approaches to safety management. Thought paper. The Health Foundation. 2012. https://www.bl.uk/britishlibrary/~/media/bl/global/social-welfare/pdfs/non-secure/p/r/o/proactive-approaches-to-safety-management.pdf.

[22] Macrae, C. Moments of resilience: time, space and the organisation of safety in complex Sosiotechnical systems. In: Wiig, S. & Falbruch, B. editors. Exploring Resilience. A scientific Journey from Practice to Theory. Springer Open; 2019.

[23] Brennan, T. The role of regulation in Quality Improvement. The Milbank Quarterly. 1998. Vol. 76, No. 4. 10.1111/1468-0009.00111. 10.1111/1468-0009.00111 PMC2751097 9879308

[24] Walshe K (2003). Regulating healthcare: a prescription for improvement?.

[25] Healy J. Improving health care safety and quality: reluctant regulators: Routledge; 2016.

[26] Hovlid E, Frich JC, Walshe K, Nilsen RM, Flaaten HK, Braut GS.…Harthug S. Effects of external inspection on sepsis detection and treatment: a study protocol for a quasiexperimental study with a stepped-wedge design. BMJ Open; 2017;7:e016213. doi: 10.1136/bmjopen-2017-016213. 10.1136/bmjopen-2017-016213 PMC5589010 28877944

[27] Schaefer C, Wiig S (2017). Strategy and practice of external inspection in healthcare services – a Norwegian– a Norwegian comparative case study. Safety in Health.

[28] Berg, S. H, Akerjordet K., Ekstedt, M. & Aase, K. Methodological strategies in resilient health care studies: an integrative review. Saf Sci 2018; 110:300–312. 10.1016/j.ssci.2018.08.025.

[29] Macrae C, Wiig S. Resilience: from practice to theory and Back again. In: Wiig, S. & Falbruch, B. editors. Exploring Resilience. A scientific Journey from Practice to Theory. Springer Open; 2019.

[30] Macrae, C. Reconciling regulation and resilience in health care. In: Hollnagel, E., Braithwaite, J., Wears, R.L. editors. Resilient Health Care. Ashgate; 2013.

[31] Bal R, Stoopendaal AM, van de Bovenkamp H. Veerkracht en veiligheid: hoe kan regulering van de zorg daaraan bijdragen? [Resilience and patient safety: how can health care regulations contribute?]. Ned Tijdschr Geneeskd. 2015;159:A9614. 26732218

[32] Stoopendaal A, de Bree M, Robben P (2016). Reconceptualizing regulation: formative evaluation of an experiment with system-based regulation in Dutch healthcare. Evaluation.

[33] Anderson JE, Ross AJ, Macrae C, Wiig S. Defining adaptive capacity in healthcare: A new framework for researching resilient performance Applied Ergonomics 87 (2020) 103111. 10.1016/j.apergo.2020.103111 32310111

[34] Regulation on management and quality improvement in the healthcare services*.* In Norwegian: Forskrift om ledelse og kvalitetsforbedring i helse- og omsorgstjenesten. FOR-2016-10-28-1250. Oslo: Ministry of Health and Care Services; 2016.

[35] Norwegian Board of Health Supervision. In Norwegian: Tilsynsmelding 2014. In English: The Annual Supervision Report 2014. Oslo: The Norwegian Board of Health Supervision; 2015. https://www.helsetilsynet.no/globalassets/opplastinger/Publikasjoner/tilsynsmelding/tilsynsmelding2014.pdf/. Accessed 8 July 2020.

[36] NOU 2015:11. In Norwegian: Med åpne kort. Forebygging og oppfølging av alvorlige hendelser i helse- og omsorgstjenestene. In English: White Paper 2015:11. Departementenes sikkerhets- og serviceorganisasjon. Oslo: Informasjonsforvaltning; 2015. https://www.regjeringen.no/contentassets/daaed86b64c04f79a2790e87d8bb4576/no/pdfs/nou201520150011000dddpdfs.pdf. Accessed 8 July 2020.

[37] Norwegian Ministry of Health and Care Services. In Norwegian: Høringsnotat. In English: Hearing Memorandum. Oslo: Ministry of Health and Care Services; 2015.

[38] Norwegian Ministry of Health and Care Services. Oslo: Ministry of Health and Care Services; 2019. https://www.regjeringen.no/en/dep/hod/id421/. Accessed 8 July 2020.

[39] Johannesen DTS, Wiig S. Why adopt ISO 9001 certification in hospitals? A case study of external triggers and sensemaking in an emergency department in Norway. Safety in Health. 2017;3:7; doi:10.1186/s40886-017-0058-5.

[40] Wiig S, Ree E, Johannessen T (2018). Improving quality and safety in nursing homes and home care: the study protocol of a mixed-methods research design to implement a leadership intervention. BMJ Open.

[41] SSB. In Norwegian: Statistikkområde. Helse: Helsetjenester. In English: Statistics. Health: Health Services. 2019. https://www.ssb.no/helse?de=Helsetjenester. Accessed 8 July 2020.

[42] Engen OAH, Kruke BI, Lindøe PH, Olsen KH, Olsen OE, Pettersen KA. Perspektiver på samfunnssikkerhet. Oslo: Cappelen Damm; 2016.

[43] Hood, C., Rothstein, H. & Baldwin, R. The Government of Risk: Understanding Risk Regulation Regimes. Oxford University Press; 2001.

[44] Rasmussen J (1997). Risk management in a dynamic society: a modelling problem. Saf Sci.

[45] Braithwaite, J. The Essence of Responsive Regulation. UBC Law Review. 2011 Vol. 44:3 475–520.

[46] Vincent C, Amalberti R. Safer Healthcare. Strategies for the Real World. 1.ed. Springer; 2016. DOI 10.1007/978-3-319-25559-0_7. 29465922

[47] Nordrum JCF (2019). Bedre Regulering? Årsak-Virkningsanalyser i Norsk Reguleringsprosess.

[48] Lindøe PH, Kriingen J, Braut GS. Risiko og tilsyn. Risikostyring og rettslig regulering. 2. Utgave. Oslo: Universitetsforlaget; 2015.

[49] Furniss D, Barber N, Lyons I (2014). Unintentional non-adherence: can a spoon full of resilience help the medicine go down?. BMJ Quality Safety.

[50] Hollnagel E, Woods DD, Leveson N (2006). Resilience engineering: concepts and precepts.

[51] Hollnagel E (2014). Safety-I and safety-II.

[52] Wiig S, Falbruch B. Exploring Resilience. A scientific Journey from Practice to Theory. Springer Open; 2019.

[53] Kyriakidis M, Dang VN. Human performance, levels of service and system resilience. In: Wiig, S. & Falbruch, B. editors. Exploring Resilience. A scientific Journey from Practice to Theory. Springer Open; 2019.

[54] Berg SH, Aase K. Resilient characteristics as described in empirical studies on health care. In: Wiig, S. & Falbruch, B., editors. Exploring Resilience. A scientific Journey from Practice to Theory. Springer Open; 2019.

[55] Anderson JE, Ross AJ, Back J (2016). Implementing resilience engineering for healthcare quality improvement using the CARE model: a feasibility study protocol. Pilot Feasibility Stud.

[56] Yin, R. K. Case Study Research. Design and Methods. SAGE Publications; 2014 (:88).

[57] Norwegian Ministry of Health and Care Services. In Norwegian: Lederansvar i sykehus Rundskriv I-2/2013. In English: Circula on management in hospitals. Oslo: Norwegian Ministry of Health and Care Services; 2013.

[58] Norwegian Ministry of Health and Care Services. In Norwegian: Oppdrag til Helsedirektoratet om å utarbeide utkast til felles forskrift for internkontroll og systematisk arbeid med kvalitetsforbedring og pasient- og brukersikkerhet i helse- og omsorgstjenesten. In English: Assignment letter of drafting a new regulation. Oslo: Ministry of Health and Care Services; 2013.

[59] Norwegian Directorate of Health. In Norwegian: Prosjektbeskrivelse for utkast til felles forskrift. In English: Project plan. Oslo: Norwegian Directorate of Health; 2013.

[60] Norwegian Directorate of Health. In Norwegian: Invitasjon til innspill til Helsedirektoratets arbeid med ny felles forskrift for internkontroll og systematisk arbeid med kvalitetsforbedring og pasient- og brukersikkerhet i helse- og omsorgstjenesten. In English: Invitation to give input to NDH’s draft. Oslo: Norwegian Directorate of Health; 2014.

[61] Norwegian Directorate of Health. In Norwegian: Utkast til høringsnotat. In English: Draft of the Hearing Memorandum. Oslo: Norwegian Directorate of Health; 2014.

[62] Norwegian Ministry of Health and Care Services. In Norwegian: Høring - Forskrift om styringssystem i helse- og omsorgstjenesten. In English: *Hearing*. Oslo: Ministry of Health and Care Services; 2016. https://www.regjeringen.no/no/dokumenter/horing%2D%2Dforskrift-om-styringssystem-i-helse%2D%2Dog-omsorgstjenesten/id2459663/.

[63] Norwegian Board of Health Supervision. In Norwegian: Høringsuttalelse. In English: Hearing Comment. Oslo: Norwegian Board of Health Supervision; 2016.

[64] Norwegian Directorate of Health. In Norwegian: Høringsuttalelse. In English: Hearing Comment. Oslo: Norwegian Directorate of Health; 2016.

[65] Norwegian Ministry of Health and Care Services. In Norwegian: Forskrift om ledelse og kvalitetsforbedring i helse- og omsorgstjenesten. Kongelig resolusjon. In English: The Prerogative. PRE-2016-10-28-1250 Oslo: Ministry of Health and Care Services; 2016.

[66] Norwegian Directorate of Health. In Norwegian: Veileder til forskrift om ledelse og kvalitetsforbedring i helse- og omsorgstjenesten. In English: Guidelines to Regulation on management and quality improvement in the healthcare services. Oslo: Norwegian Directorate of Health; 2017.

[67] Eckhoff T (2001). Rettskildelære.

[68] Miles MB, Huberman AM, Saldana J. Qualitative Data Analysis. A Methods Sourcebook. Sage Publications; 2014.

[69] Hsieh H-F, Shannon SE. Three Approaches to Qualitative Content Analysis. Qual Health Res. 2005;15: No. 9; 10.1177/1049732305276687. 10.1177/1049732305276687 16204405

[70] Pattaro E (2005). A treatise of legal philosophy and general jurisprudence.

[71] Blaikie N (2010). Designing social research.

[72] Tong A, Sainsbury P, Craig J. Consolidated criteria for reporting qualitative research (COREQ): a 32-item checklist for interviews and focus groups. Int J Qual Health Care. 2007;19(6):349–357; 10.1093/intqhc/mzm042. 10.1093/intqhc/mzm042 17872937

[73] Graneheim UH, Lundman B. qualitative content analysis in nursing research: concepts, procedures and measures to achieve trustworthiness. Nurse Educ Today. 2004;24(2):105–12. 10.1016/j.nedt.2003.10.001. 10.1016/j.nedt.2003.10.001 14769454

[74] Black: The Law Dictionary. 2019. https://thelawdictionary.org/functional-regulation/2019/. Accessed 8 July 2020.

[75] Deming, W. E. Out of the crisis, Massachusetts Institute of Technology Center for Advanced Engineering Study; 1986.

[76] Norwegian Ministry of Health and Care Services. In Norwegian: Instruks for Helsedirektoratet. Fastsatt av Helse- og omsorgsdepartementet. In English: Instructions for the Norwegian Directorate of Health. Set by the Norwegian Ministry of Health and Care Services. Oslo: Norwegian Ministry of Health and Care Services; 2018.

[77] Dekker S (2014). The bureaucratization of safety. Saf Sci.

[78] Wiig S, Aase K, Bal S. Reflexive spaces: Leveraging resilience into healthcare regulation and management. J Patient Saf. January 31, 2020 - Volume Publish Ahead of Print - Issue - 10.1097/PTS.0000000000000658. 10.1097/PTS.0000000000000658 PMC8612922 32011428

[79] Furniss et al. Using FRAM to explore sources of performance variability in intravenous infusion administration in ICU: A non-normative approach to systems contradictions. Appl Ergon. 2020;86:103113. 10.1016/j.apergo.2020.103113 32342897

[80] Wears RL, Hollnagel E, Braithwaite J. Resilient health care, volume 2: the resilience of everyday clinical work. CRC Press; 2015.

[81] Lindøe P, Baram M, Renn O (2014). Risk governance of offshore oil and gas operations.

[82] Woods DD (2009). Escaping failures of foresight. Saf Sci.

[83] Woods DD (2015). Four concepts for resilience and the implications for the future of resilience engineering. Reliab Eng System Saf.

[84] Heimer C (2013). A. Resilience in the middle. Contributions of regulated organizations to regulatory success. Ann Am Acad Pol Soc Sci.

[85] Vincent C, Amalberti R (2015). Safety in healthcare is a moving target. BMJ Qual Saf.

[86] Macrae, C. Regulating resilience? Regulatory work in high-risk arenas. In: Hutter, B. M. editor. Anticipating Risks and Organising Risk Regulation. Cambridge University Press; 2010.

[87] Hutter, B. M. Understanding the New Regulatory Governance: Business Perspectives. Law & Policy. 2011;33(4). 10.1111/j.1467-9930.2011.00346.x.

[88] Macrae, C. Early warnings, weak signals and learning from healthcare disasters. BMJ Qual Saf. 2014; 23:440–445. 10.1136/bmjqs-2013-002685. 10.1136/bmjqs-2013-002685 24599729

[89] Øyri S, Wiig S. Regulation and resilience at the macro-level healthcare system – a literature review. Proceedings of the 29^th^ European safety and reliability conference 2019. Editors: Michael Beer and Enrico Zio doi: 10.3850/978-981-11-2724-3_ 0075-cd.

[90] Van de Bovenkamp, H. M, Stoopendaal, A. & Bal, R. Working with layers: the governance and regulation of healthcare quality in an institutionally layered system. Public Policy Adm 2017;32:45–65. 10.1177/0952076716652934. 10.1177/0952076716652934 PMC5447897 28596640

[91] Wiig S, Lindøe P (2009). Patient safety in the interface between hospital and risk regulator. J Risk Res.

[92] Bromley, P. & Powell, Walter W. From Smoke and Mirrors to Walking the Talk: Decoupling in the Contemporary World, Acad Manag Ann. 2012;6:1, 483–530. 10.1080/19416520.2012.684462.

[93] de Bree M, Stoopendaal A. De- and recoupling and public regulation. Organ Stud. 2018; 10.1177/0170840618800115.

[94] Wiig S, Schibevaag L, Zachrisen RN, Hannisdal E, Anderson J, Haraldseid-Driftland C. Next-of-Kin Involvement in Regulatory Investigations of Adverse Events That Caused Patient Death: A Process Evaluation (Part II The Inspectors' Perspective). J Patient Saf, Publish Ahead of Print () October 22, 2019. 10.1097/PTS.0000000000000634. 10.1097/PTS.0000000000000634 PMC8612908 31651541

[95] Simon MD (2018). Compliance and high reliability in a complex healthcare organization. Front Health Serv Manag.

[96] Chatburn E, Macrae C, Carthey J (2018). Measurement and monitoring of safety: impact and challenges of putting a conceptual framework into practice. BMJ Qual Saf.

[97] Bowen, G. A. Document Analysis as a Qualitative Research Method. Qual Res J. 2009; Vol. 9 No. 2, pp. 27–40; :29. 10.3316/QRJ0902027.

[98] Malterud, K., Siersma, V. D, Guassora, A. D. Sample size in qualitative interview studies: guided by information power. Qual Health Res. 2016;13:1753–1760. 10.1177/1049732315617444. 10.1177/1049732315617444 26613970

[99] Labaree, R. Organizing your social sciences research paper: limitations of the study*.* 2009. https://libguides.usc.edu/writingguide/limitations. Accessed 8 July 2020.

[100] Iflaifel MHM, Lim R, Ryan KM, Crowley C. Resilient Health Care: a systematic review of conceptualisations, study methods and factors that develop resilience. BMC Health Serv Res. (In Review) 2020. 10.21203/rs.2.16286/v4. 10.1186/s12913-020-05208-3 PMC7165381 32303209

