# Supplementary material for: Exploring links between resilience and the macro-level development of healthcare regulation- a Norwegian case study
Source: BMC Health Serv Res. 2020 Aug 18;20:762. doi: 10.1186/s12913-020-05513-x (PMC7433050; doi:10.1186/s12913-020-05513-x)
Supplement: Supplementary file 1 — Additional file 1. “Interview guide”. A semi-structured interview guide based on theoretical perspectives on resilience and risk regulation regimes and based on information retrieved from the documents. The topics included: rationale, experiences of stakeholder involvement and information processes, expectations regarding implementation and capacity for regulatory flexibility. [file 12913_2020_5513_MOESM1_ESM.docx]

### Interview guide

- Please introduce yourself (name, educational- and professional background, current position).
- What was the rationale for the government’s adaptation of the Internal Control Regulations into the new Quality Improvement Regulation explicitly targeting managers?
- What is quality improvement to you?
- How does the management focus represent a necessary change for quality improvement in healthcare?
- How do you expect the new Quality Improvement Regulation to facilitate quality improvement, patient safety (and safe patient treatment), and thereby improving hospital performance?
- In what ways did you (the government) involve clinicians within hospital management during the development of the new Quality Improvement Regulation?
- How did you communicate and inform about the regulatory adaptations to the hospitals?
- What was the information strategy when the Quality Improvement Regulation went into effect?
- In retrospect, what would you have done differently in terms of development and implementation of the Quality Improvement Regulation?
- Looking at internal control as government control of hospitals self-regulation of risk: what were the reasons behind such a performance-based regulatory strategy (specifying certain goals, but details on how to achieve these goals are left out)?
- Do you consider the new Quality Improvement Regulation to facilitate flexibility and adaptation for hospital managers improvement work, if so, how?
- What expectations do you have towards hospital managers knowledge- and competences within systems for safety and quality improvement?
- What do you consider to be the advantages with the new Quality Improvement Regulation?
- What kind of disadvantages with the regulatory framework do you expect?
- What feedback, positive or negative, have you received in the aftermath of introducing the Quality Improvement Regulation?
- Looking ahead – let’s say five years from now, what sort of changes does the government expect the new Quality Improvement Regulation to contribute to?
- Is there anything we have not talked about yet, that you feel is important to mention?
